# The Efficient Heritage of the Craftsmanship Spirit in China: A Configuration Effect of Family Motivation and Organizational Learning

**DOI:** 10.3389/fpsyg.2022.807619

**Published:** 2022-04-08

**Authors:** Guodong Chen, Jingqing Du, Ri Shan, Liwei Lu, Xiaoyan Mao

**Affiliations:** ^1^Business College, Taizhou University, Taizhou, China; ^2^School of Art and Design, Taizhou University, Taizhou, China; ^3^School of Foreign Languages, Taizhou University, Taizhou, China

**Keywords:** family motivation, craftsmanship spirit, organizational learning, fsQCA, the institution-led path, the motivation-driven interpretation-led path

## Abstract

In China, cultivation of the craftsmanship spirit is strongly advocated, but little attention is devoted to whether and how “working for the family” promotes heritage of this spirit. A configuration model of family motivation and organizational learning is proposed and expounded. Fuzzy set qualitative comparative analysis (fsQCA) was used to further explore the conditional configuration. The results show that the fitting family motivation to organizational learning is important for promoting heritage of the craftsmanship spirit. There are two paths that promote efficient heritage of this spirit: the institution-led path and the motivation-driven interpretation-led path. For the institution-led path, when apprentices have strong institution learning ability, the anterior-cause condition involves low family motivation, but this factor plays a weak role in promoting efficient heritage of the craftsmanship spirit. In a configuration consisting of intuition, interpretation, integration and institution, family motivation becomes irrelevant, which explains the phenomenon that organizational learning behavior reinforcement leads to a lack of family motivation. For the motivation-driven interpretation-led path, even without regular and institutionalized learning behaviors in the organization, the core conditions of high family motivation and high explanation learning, together with low intuition learning and low integration learning, will promote efficient heritage of the craftsmanship spirit.

## Introduction

Ancient China is famous for its “four great inventions.” Craftsmanship has been passed down from generation to generation, and many masterpieces have been created. Craftsmen have cultivated and inherited the family-based traditional Chinese craftsmanship spirit, which has been incorporated with more innovative elements and is being transformed toward industrialization and marketization in modern industrial civilization. However, the pragmatic aspect of short-term income poses a severe challenge to the heritage of the craftsmanship spirit, generating a deficit in the spirit in current Chinese industrial culture. Chinese tourists’ purchase of rice cookers, toilet seats and other commodities abroad reflects the deficiency of the “craftsmanship spirit” in the Chinese manufacturing industry. Since 2016, the term “craftsmanship spirit” has frequently appeared in central government policy documents, and the cultivation of the craftsmanship spirit has become the focus of many scholars (Luo et al., 2020; Wang, 2020; Jin, 2021). Craftsmanship spirit is a type of professional spirit; its core content is workers’ unremitting pursuit of excellence in their own work (Wang, 2020; Jin, 2021). Zhang noted that the craftsmanship spirit is based on precision, patience and perfection (Zhang and Cerdin, 2020). The relationship between family background and work status is often discussed (Su and He, 2020), However, the above literature ignores the effect of working for the family on the development of individual spiritual quality. To examine the relationship between family and the craftsmanship spirit, it is necessary to adhere to the family-oriented thinking and incorporate the current concept of matter, so as to fully understand the craftsmanship spirit and its heritage.

With continuous improvements in prosocial motivation theory (Grant, 2008), the academic circle has started to use prosocial motivation to interpret employees’ work status and mental outlook, which has prompted an understanding on the effect of pro-family motivation on work performance (Bolino and Grant, 2016). Prosocial motivation is a type of willingness that allows an individual to devote themselves to others (Tian et al., 2021), showing a positive effect in promoting creativity and cultivating persistence (Grant, 2008). Miller et al. (2012) explained the logic of this motivation in detail, i.e., producing behavioral outcomes through emotional and cognitive processes. Both social cognitive theory (Bandura, 2001) and the motivation-opportunity-ability (MOA) model (Blumberg and Pringle, 1982) agree that an individual’s motivation and behavior affect and interact with each other (Nijstad and De Dreu, 2012). Organizational learning is a type of individual behavior pattern and shows complex interaction with prosocial motivation, and in the presence of strong motivation, organizational learning behavior is naturally strengthened (Grant and Berry, 2011). Family motivation, a special case of prosocial motivation (Menges et al., 2016), is the willingness to work to support family life and thus plays an important role in increasing work productivity (Shao et al., 2017; Zhang et al., 2019) and job loyalty and decreasing turnover tendency (Tariq and Ding, 2018). Similarly, it also interacts with organizational learning. From the perspective of positive organizational behavior, pro-family learning objectives improve persistence regarding learning tasks, while scholars also recognize the view that “to learn better, give back to the family”; after all, personal motivations are often rooted in the family environment and are profoundly influenced by family members (Barreto et al., 2014). From the perspective of negative organizational behavior, family motivations can also bring unintended consequences, including disloyal behaviors, and even the violation of basic social and organizational ethics for the benefit of family (Liu et al., 2020). These research results can explain China’s lack of craftsmanship spirit today. To make their family rich quickly, people can act disloyally in work and even form a short-term profit-oriented work attitude and exhibit speculative, immoral and unethical behaviors. In the apprenticeship system that is born out of the family structure, working for the family is one of the reasons craftsmen insist on passing on skills (Nielson et al., 2001). Although in the industrial age apprenticeship has evolved and its members are not limited to family members (Son, 2016), the craftsman’s view of family interests has not changed substantially. In summary, in the workplace of apprenticeship, family motivation can prompt craftsmen to maintain their craft, but the internal mechanism by which family motivation promotes the heritage of the craftsmanship spirit is still unclear. Moreover, given that family motivation and organizational learning behavior affect each other, does the combination of the two affect the heritage of the craftsmanship spirit? If yes, then how? The mechanism by which the craftsmanship spirit is inherited is unclear, and as a configuration problem, it cannot be solved through traditional regression analysis; fuzzy set qualitative comparative analysis (fsQCA) based on the logic of abduction should be used (Du and Kim, 2021).

Thus, the apprenticeship system is selected to comprehensively examine the configuration effect of the combined factors of craftsman’s family motivation and organizational learning in promoting the efficient heritage of the craftsmanship spirit. Unlike previous studies, this study provides more detailed interpretations in the following areas: first, it validates that family motivation and organizational learning constitute a complete cognitive learning system, which is an important condition for the heritage of the craftsmanship spirit of craftsmen; second, it challenges the conclusion of previous studies that use family motivation as a single important element; instead, it describes a variety of ways of promoting the heritage of the craftsmanship spirit from the perspective of fitting family motivation and organizational learning; and third, it examines the complementary relationship between family motivation and organizational learning behavior from the perspective of reverse inhibitory factors.

## Theoretical Framework

With the development of the times and the change in concepts, craftsmen have been given new historical connotations, and in the new era, the industrial boundary surrounding craftsmen has been weakened, and occupational restrictions have been broken; therefore, craftsmen include scientists in research institutions, skilled personnel in enterprises and personnel in public institutions. The stereotype of a craftsman reflects the urgency of transformation in the study of the modern craftsman and the necessity of attaching importance to the prosocial motivation of craftsmen in the new era when analyzing their perfectionism and perseverance.

**FIGURE 1:**
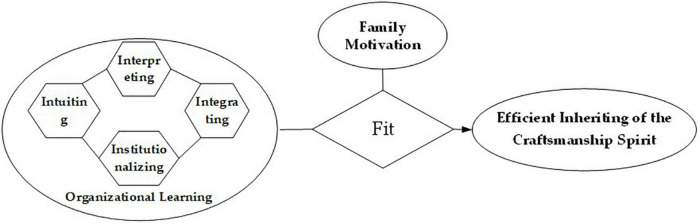
Conceptual model.

Social cognitive theory (Bandura, 1986, 2001) discussed the dynamic relationship among intrapersonal influences, the behavior individuals engage in and the environmental forces impinge on. The three parties interacted as both cause and effect, and there was a two-way interaction between every two parties, which jointly promoted the emergence of another behavior. Bandura (1986) also discussed observational learning in detail in social cognitive theory, which was used to explain the problem of result reinforcement and behavior correction in the process of individual learning, and emphasized the importance of learning behavior to the results. According to social cognitive theory, the apprentice’s craftsmanship spirit inheritance behavior will be influenced by intrapersonal influences and the behavior individuals engage in. Based on the above theory, combined with organizational learning 4I framework (Crossan et al., 1999), this paper puts forward the research framework fitting family motivation to organizational learning (as shown in Figure 1), and shows that the efficient inheritance of apprentice’s craftsmanship spirit is the result of the interaction between family motivation and organizational learning such as intuition, interpretation, integration and institutionalization. It is a complete cognitive learning system.

In recent years, family motivation and work input have been extensively investigated (Grant and Berry, 2011; Menges et al., 2016; Zhang et al., 2019). In the presence of strong family motivation, craftsmen connect important family values and strengthen the self-awareness of family responsibility and role model (Grant and Berry, 2011), which motivate them to work hard and consistently. In fact, by regarding family roles as an important part of identity, craftsmen devote more time to work (Rothbard and Edwards, 2003), which is more conducive to the cultivation of their craftsmanship spirit. According to the theory of prosocial motivation, the joint effect of motivation and behavior lead to outcomes (Su and He, 2020). In the study of craftsman behavior, the heritage of the craftsmanship spirit is inseparable from the influence of the master on the craftsman’s intrinsic characteristics as well as from the craftsman’s talent in “sensed” learning (Nielson et al., 2001), which, in teams with high prosocial motivation, is more conducive to creating conditions for learning from each other; therefore, a craftsman’s reflection on work can be more thorough because the learning behavior has become a key link in inheriting the craftsmanship spirit in the apprenticeship system. Therefore, when analyzing the craftsman’s heritage of the craftsmanship spirit, two factors should be considered, i.e., the craftsman’s family motivation, which is essentially the willingness to work for family wellbeing, and the craftsman’s organizational learning behavior, which is manifested in the craftsman’s cognition and understanding, interpretation and dialog, knowledge internalization, and behavioral norms, etc.

Based on the above analyses, a configuration model is proposed, as shown in Figure 1.

### Craftsman’s Family Motivation

Everyone has a motivation to work. Family motivation is the willingness to work for family (Menges et al., 2016), which is the main work motivation of most people. Family motivation is a type of eudemonism, which emphasizes the importance and value of assuming family responsibility (Grant and Berry, 2011) and exerting effort for the wellbeing of family members in terms of growth, health, psychology and material providence. Among them, the interests of family are the most powerful source of value. Hence, family motivation is a family-associated prosocial motivation.

Additionally, the influence of family motivation on a craftsman’s work is a “double-edged sword,” affecting the heritage of the craftsmanship spirit in different aspects. On the one hand, when growing into various leadership roles, such as supervisor, core worker, and master, a craftsman could be given special role requirements by an organization as well as high expectations. The organization hopes that craftsmen work whole-heartedly for the organization, a hope that conflicts with the will of family members who want more family time (Lapierre et al., 2012) and thus leads to negative work performance (Stollberger et al., 2019). In addition, a core connotation of family motivation is that craftsmen regard work as a means of family support, which leads to the pursuit of short-term economic gain and ultimately undermines their sustained investment in work (Menges et al., 2016). On the other hand, in traditional Chinese families, the family concept of “be proud when working” prevails and fosters the notion that work is a means of enhancing family status and reputation; therefore, hard work essentially manifests as an individual’s family responsibility (Greenhaus and Powell, 2006). When working for the benefit of family members, craftsmen strengthen their willingness to work hard for a long time due to the deep blood tie to beneficiaries (Grant, 2007).

### Craftsman’s Organizational Learning

In an apprenticeship system, craftsmen engage in learning practices involving internalization, sharing and routinization, whose learning mechanism follows the 4I framework of organizational learning proposed by Crossan et al. (1999), which has implications for analyzing the heritage of the craftsmanship spirit. The 4I framework includes four processes: intuition, interpretation, integration and institutionalization. Crossan emphasized that not all organizational learning could have the four processes and that organizational learning could neither strictly start from nor end at a certain process (Grant, 2007).

Specifically, the intuition is a subconscious process, and the craftsman’s intuition involves “predicting the outcome of behavior, setting goals, motivating oneself, and learning by observing the behavior of the master” (Bandura, 1988; Wang et al., 2017). Intuition occurs at the craftsman’s personal level. When making the leap to the team level with the master as the core, a craftsman shares his/her experience and skills with others in a metaphorical way, so that the craftsman’s quality and skills can be elevated to a higher level.

Interpretation is related to the refinement of intuitive insight, and as Polanyi said, “comprehension requires an individual to have perceptions of different difficulties.” In the learning process of interpretation, a craftsman uses language to eliminate differences with the master, forms an effective dialog process with team members, including the master, and establishes exchange and learning activities in the forms of seminars, lectures, and meetings, enabling the craftsmanship spirit to propagate from the individual to the team.

The integration is a collective cognitive process with the craftsman as the core, through which the craftsmanship spirit permeates throughout the team. Thus, the mutual adjustment and collaboration of group members are particularly important. Through interactions with others, the craftsman shares knowledge while generating new ideas (Aponte and Zapata, 2013).

The institutionalization takes place at the organizational level. Organizations have their own framework, inertia, and strategy. When embedded in the organization, the apprenticeship work model provides organizational guarantees for the heritage of the craftsmanship spirit; therefore, spontaneous individual and team learning is no longer the mainstream style (Grant, 2007), and compliance with organizational norms becomes the basic law for the presence of apprenticeship. However, organizational norms are constantly adjusted as the organization develops, and therefore, the organizational diagnostic system plays the core role. Once adjusted, the organization must be present in a certain institutional framework for a long time until the next change occurs. Such a stable organizational and institutionalized learning guarantees the heritage of the craftsmanship spirit.

### Fitting Craftsman’s Family Motivation to Organizational Learning

In the apprenticeship system, craftsmen are the core of the heritage of the craftsmanship spirit. They receive training, abide by the rules, learn the skills and characters of the master, and then pass the skills and characters to their own apprentices. From the perspective of family motivation, the heritage of the craftsmanship spirit can be considered from the following three aspects. First, the expectations of family members will enhance craftsman’s family motivation. When a craftsman’s work can benefit the family and elevate the family in terms of reputation and income, family members could be proud of the craftsman’s skills and work, which, when perceived by the craftsman, could make him/her more passionate about, determined and committed to work (Greenhaus and Powell, 2006) and to continue his/her work and further form a correct work ethic. Second, the family responsibility will enhance craftsman’s family motivation as well, which is another reason that motivates a craftsman to improve his/her skills. When feeling that family members depend on him/her, the craftsman can strengthen his/her sense of family responsibility (Morrison and Phelps, 1999; Nijstad and De Dreu, 2012), which makes him/her work hard and persistently to elevate the family status. Menges et al. (2016) described this phenomenon in terms of meaningfulness and argued that family motivation creates a sense of meaningfulness and that employees work more persistently and more smartly by enhancing their enthusiasm toward work. Third, the learning behavior is inspired by family motivation. Previous studies have shown that family support policies can promote employees’ work-related learning behavior (Loon et al., 2012), resulting in a more enthusiastic working state (Seligman and Csikszentmihalyi, 2000), which explains the two dimensions that stimulate more work activity, i.e., family motivation and learning behavior. The more general logic is that the combination of family motivation and learning behavior is the basis for improving a craftsman’s cognitive and professional abilities, which are the key elements in forming the “knowledge difference” between the master and the apprentice. In the apprenticeship system, it is the precisely the “knowledge difference” between the master and the craftsman that leads to the heritage of the craftsmanship spirit.

Whether family motivation can promote the heritage of the craftsmanship spirit also depends on the craftsman’s organizational learning behavior. According to Amabile’s (1988) componential theory of creativity, to have creativity, employees require a long and self-reinforcing learning so that they can acquire creativity-related knowledge, skills and characters. Similarly, the cross-level emergence theory of human capital resources also proves this view, i.e., the organization-level constructs and phenomena originate from and are affected by low-level psychological and behavioral mechanisms (Abell et al., 2008), and the emergence of a phenomenon is the result of the members’ continuous interactions to adapt to the situation (Ployhart and Moliterno, 2011). The emergence of the craftsmanship spirit is a process that organizes the enlightenment of first-line craftsmen, the internalization of master and apprentice, sharing among mid-level organizations and departments, and commitment and recognition of leadership. The emergence and transmission process of the craftsmanship spirit, as a special human capital resource, cannot do without the master’s motivation factors nor the apprentice’s learning elements. Therefore, for craftsmen, family motivation plays an important role in promoting the heritage of the craftsmanship spirit. However, to facilitate the passing on of the craftsmanship spirit, family motivation needs to fit the organizational learning behavior, in which the configuration effect of the two is more crucial. There is a multiple fitting relationship between family motivation and organizational learning behavior.

Family motivation is one of the forces that drive a craftsman to engage in work. As a learner, craftsmen can interpret and learn the knowledge and character of the master from intuition, complete knowledge integration in a series of interactive behaviors and inherit the craftsmanship spirit by means of institutionalization. However, craftsman’s initiative will be affected by organizational learning as well. Noe (1988) examined craftsman’s initiative and noted that a craftsman’s personality, gender, interactive relationship, and other factors have a certain influence on the craftsman’s work acceptance and that the craftsman’s active learning can be recognized by the master. de Janasz and Godshalk (2013) verified that E-mentoring was positively associated with protégés’ learning. The higher the frequency of interactive learning with tutors was, the better the learning effect would be. Ghosh (2014) argued that a high learning goal orientation is beneficial for promoting a craftsman’s active learning behavior while enriching the master-apprentice relationship.

Family motivation with high family goals based on a sense of family responsibility and role model, as a learning goal orientation, can reduce a craftsman’s self-interest oriented immoral behavior (Menges et al., 2016) and make the craftsman explore new knowledge, develop new skills, and take on new tasks without fear. Certainly, individual learning goal orientation with family as the core and starting point often fits subsequent individual active learning, and the process of passing on the craftsmanship spirit from master to craftsman includes a series of dynamic processes in which the craftsman establishes correct goal orientation and engages in organizational learning. The 4I framework of organizational learning explains the learning behavior of craftsmen from the individual level to team level; it affirms not only the process of learning behavior but also the combination of learning willingness and learning behavior while showing the possibility of combining learning motivation and learning behavior. In the field of organizational learning, which is based on the dualistic relationship between master and apprentice, the academic circle lacks investigations of the combination of learning motivation and behavior; after all, organizational learning processes are very diverse (Crossan et al., 2011). In summary, in terms of promoting the heritage of the craftsmanship spirit, fitting family motivation to organizational learning presents a variety of combinations, each of which contains a certain theoretical value.

### Relationship Between the Lack of Family Motivation and the Strengthening of Organizational Learning Behavior

Various scholars, e.g., Menges et al. (2016) and Zhang et al. (2019), described the reverse effect of family motivation from different perspectives, i.e., family motivation can also inhibit improvements in work performance and creativity. Zhang et al. (2019) even argued that family motivation originated from family role models and that the establishment of a special family identity can result in more autonomy, leading to different outcomes. Under different situations, family motivation is not critical to work outcomes, especially when other factors play greater roles. According to a study on social learning theory, leaders who set an example through their own learning behavior can stimulate employees’ prosocial motivation (Crossan et al., 1999; Su and He, 2020) and family motivation. In the context of the apprenticeship system, craftsmen can subtly comprehend the master’s learning habits and behaviors and use the example of the master to pass on learning behaviors to future apprentices. In this process, learning behavior plays a leading role and thus a greater role in promoting the heritage of the craftsmanship spirit. The reinforcement of learning behavior could lead to a loss of family motivation. In other words, when organizational learning behavior plays a greater role in promoting the efficient heritage of the craftsmanship spirit, family motivation becomes irrelevant.

## Materials and Methods

To effectively analyze the mechanism by which the craftsmanship spirit is inherited, it is necessary to rationally select research sites and objects. Given that the longer is a craftsman’s work experience, the more profound the craftsman’s understanding of family and work, in this study, craftsmen within the new era with more than 10 years of apprentice experience were selected as subjects for a questionnaire survey, and the questionnaire survey and an in-depth interview were conducted at the workplace of the apprenticeship.

The fsQCA was adopted in this study. In 1987, Ragin proposed the research paradigm of qualitative comparative analysis (QCA). In his subsequent works, he introduced in detail three methods, i.e., crisp set qualitative comparative analysis (csQCA), multivalue set qualitative analysis (mvQCA), and fsQCA (Ragin, 2008; Rihoux and Ragin, 2009). The QCA method describes a multiple concurrent causality that is non-linear and non-probabilistic and emphasizes that different combinations of antecedent conditions can lead to the same outcome, and the combination of conditions is complex and diverse. QCA is a useful tool for analyzing the complexity of causality and used to evaluate very complex configuration causes and generate different combinations of antecedents for the same outcome.

The reasons for using fsQCA to study the configuration problem of craftsman learning in an apprenticeship system are as follows. (1) Mainstream quantitative analyses have been focused on the analysis of net effects at the variable level and overlooked solving the problem of combining interdependent antecedents (Porfírio et al., 2019). Multiple factors prompt the heritage of the craftsmanship spirit, and the outcome of the multiprocess joint action of craftsmanship mentality and behavior and the combination effect of condition factors can be analyzed through fsQCA. (2) The conditions for the efficient heritage of the craftsmanship spirit are multiple, concurrent and interdependent rather than independent, to which QCA, as a configuration perspective, provides a better solution. Additionally, the data collected using Likert scales are all continuous variables, and the fsQCA can solve the problems of degree changes in and membership degree of continuous variables. (3) The apprenticeship system is the best case for observing the mechanism by which the craftsmanship spirit is inherited. Successful and solid “apprenticeship system” work modes are relatively rare. Because the sample size is small, the collected data do not support traditional multivariate statistical analysis methods, such as regression analysis and structural equation modeling, but fsQCA has a unique advantage in analyzing cases with a small sample size.

### Questionnaire Design and Data Analyses

To better interpret the combined effect of family motivation and organizational learning, it is necessary to establish measurement indicators for antecedent variables (Table 1) to quantitatively analyze them. Family motivation was measured using the scale by Mengns and Zhang, which includes two core indicators of the five indicators from the perspective of craftsman and family, i.e., “the craftsman cares about and supports the family” and “The family benefits from the craftsman’s work.” The 4I framework of organizational learning was measured using the Crossan “input/outcome” variables, of which the measurement indicators of the intuition include experience, image, and metaphor, those of the interpretation include language and cognition, those of the integration process include collective knowledge, mutual adjustment, and interactive system, and those of the institutionalization include routine, diagnostic, and regular procedures.

**TABLE 1:** Calibration anchor for each variable.

Variable	Complete membership	Intersection	Complete non-membership
Family motivation (FAMOT)	4.99	4.50	4.00
Intuition (INTU)	4.99	4.72	4.51
Interpretation (INTP)	4.99	4.50	3.88
Integration (INTG)	4.91	4.70	3.98
Institutionalization (INST)	4.99	4.33	4.00
Craftsmanship spirit (GJJS)	4.99	4.58	4.00

In the heritage of the craftsmanship spirit, craftsmen play a “inherit the past and enlighten the future” role in an apprenticeship system; therefore, when measuring the heritage of the craftsmanship spirit, both the comprehensive summary of the connotations of the craftsmanship spirit and the accurate expression of “heritage” should be considered, which were measured through four indicators in this study, i.e., “the propagation of perfectionism,” “the cultivation and dissemination of lifelong pursuit of accomplishment,” “the comprehension and teaching of continuous innovation momentum,” and “the cultivation and penetration of an attitude of professionalism and responsibility.” Based on the above indicators, a survey questionnaire, written in plain and easy-to-understand language, was designed, and a five-point Likert scale was used for scoring.

A total of 32 craftsmen responded to the questionnaire survey and were interviewed, from which relevant information was also collected. First, in the unstructured face-to-face interview, various issues, such as job description, family history, work-family relationship, the influence of family attitude on work, communication with the master, and communication with the team, were included. Second, during the questionnaire survey, each respondent received instructions regarding the items on the questionnaire so that the subjects could respond based on their own perception of the work situation; each item received a score of 1, 2, 3, 4, or 5, in descending order. After collating the interview content, the questionnaires were labeled to match the interviews to facilitate the analysis. Third, information related to each craftsman’s development and cultivation, including government policy documents and regulations in the region where the craftsman lived, corporate policies and regulatory documents, etc., was collected.

### Data Aggregation

The data pertaining to the condition and outcome variables were collected using a Likert scale. Before the data analysis, the data were aggregated. The data pertaining to secondary indicators are often averaged to generate the measurement data (Sharma and Srinivas Rao, 2000; Roig-Tierno et al., 2016; Sun et al., 2020), which is effective and thus was also adopted in this study, i.e., the data reflecting the condition and outcome variables were processed using the averaging method.

### Calibration of Conditions and Outcomes

Calibration is the process of assigning a set membership score to cases (Schneider and Wageman, 2012). The calibration of a variable to a set requires a sufficient theoretical basis and external knowledge standards, and three critical values, i.e., complete membership, intersection and complete non-membership, should set up. The value range of the set membership after calibration is [0,1]. Based on the actual situation of the data and combined with the recommended value by Tosmana software, the intersection can be comprehensively determined. Du and Kim (2021) analyzed three techniques to determine which qualitative anchors to use for scale measures. Based on the percentile breakpoints, it is one of calibration to be employed when substantive knowledge about scale anchors is unavailable (De Crescenzo et al., 2020). Because the study of the craftsmanship spirit still lacks a mature theoretical system, in this study, anchors, i.e., complete membership, intersection and complete non-membership, were chosen based on the recommendations of previous studies, e.g., Fiss (2011),Andrews et al. (2016), and Son (2016). The anchor values were determined based on the 75, 50 and 25% quantiles of the sample data and are shown in Table 1.

### Necessary Condition Analysis

If a certain condition is always accompanied with the heritage of the craftsmanship spirit, then this condition is deemed a necessary condition for the heritage of the craftsmanship spirit. The necessary condition is a superset of outcomes and may be simplified in the parsimonious solution of the “logical remainder term” (Rihoux and Ragin, 2009), making the combination of conditions not comprehensive enough. Therefore, when performing configuration analysis, it is necessary to conduct necessary condition analysis on each condition first; the analysis results are shown in Table 2. In the necessary condition test of the efficient heritage, the consistency value for each condition is below 0.9, indicating that each condition is not a necessary condition for the efficient heritage of the craftsmanship spirit.

**TABLE 2:** Analysis of necessary conditions.

Condition variable	Outcome variable	
	Efficient inheritance	Non-efficient inheritance
High family motivation (fs_FAMOT)	0.814286	0.462604
Non-high family motivation (∼fs_FAMOT)	0.411538	0.688889
High intuition (fs_INTU)	0.761538	0.452524
Non-high intuition (∼fs_INTU)	0.467033	0.603516
High interpretation (fs_INTP)	0.665385	0.458796
Non-high interpretation (∼fs_INTP)	0.499451	0.697112
High integration (fs_INTG)	0.752198	0.404040
Non-high integration (∼fs_INTG)	0.448901	0.819380
High institutionalization (fs_INST)	0.849451	0.464493
Non-high institutionalization (∼fs_INST)	0.301594	0.833333

### Sufficient Condition Analysis

Sufficiency analysis assessed whether the configuration of condition is a subset of the outcome set (Crilly et al., 2012). Schneider and Wageman (2012) pointed out that the consistency level in adequacy analysis should not be below 0.75, while the frequency threshold depends on the sample size. In this study, the original consistency level value was set to 0.80, the frequency threshold was set to 1, and the proportional reduction in inconsistency (PRI) level was set to 0.70. As suggested by Fiss (2011), the configuration analysis results are presented in graphical form (core conditions are present 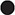 or absent 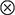, and peripheral conditions are present 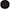 or absent 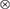; a blank space is inserted where the presence or absence of conditions does not matter) to more clearly show the importance of each condition in the combination.

Table 3 presents the four configurations that generate efficient heritage, and the consistency of each configuration as well as the consistency of each solution are greater than 0.75, the standard value for consistency. Because H1a, H1b are and H1c have the same core conditions, the three configurations are viewed as the same path for the generation of efficient heritage of the craftsmanship spirit, i.e., H1. The path with family motivation as the core condition is H2. Among the four configurations for the efficient heritage of the craftsmanship spirit, the overall consistency value is 0.771, indicating that the heritage of 77.1% of the craftsmanship spirit is at a high level, and the overall coverage rate is 0.952, i.e., all configurations can explain 95.2% of the cases.

**TABLE 3:** Configuration model of inheritance of the craftsmanship spirit.

Condition variable	Efficient inheritance				Non-efficient inheritance
		
	H1			H2	NH1
		
	H1a	H1b	H1c	H2	
Family motivation (FAMOT)	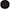	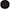		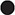	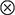
Intuition (INTU)	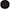		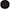	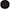	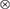
Interpretation (INTP)	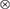	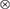	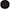	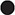	
Integration (INTG)		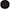	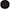	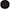	
Institutionalization (INST)	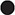	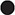	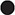		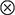
Consistency	0.960	0.876	0.989	0.969	0.956
Coverage	0.652	0.169	0.467	0.472	0.818
Unique coverage	0.181	0.033	0.040	0.046	0.818
Overall consistency	0.771				0.956
Overall coverage	0.952				0.818

*Core conditions are present 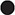 or absent 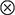, and peripheral conditions are present 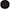 or absent 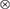; a blank space is inserted where the presence or absence of conditions does not matter.*

The theorization of configuration requires the designation of each configuration (Son, 2016), and paths H1 and H2 are, respectively, designated as institutionalization-led and motivation-driven interpretation-led configurations.

(1) Institutionalization-led configuration–In the three configurations of this path, the high level of institutionalization is the core condition. In the cases covered by the three configurations, the presence of a set of mature mentoring work rules in the company is the common feature, which includes the master selection conditions and procedures, the assessment system for the master and apprentices, incentives for the master and apprentices, craftsman promotion rules, etc. Notably, in a workplace with the institutionalization-led configuration, most cases also mention the incentive measures and welfare measures for craftsman’s family members, further confirming the importance of family motivation.

The presence of a high level of institutionalization in configuration H1a plays a central role, and the presence of non-high family motivation and non-high intuition and the absence of non-high interpretation jointly play a supporting role. H1a shows that family motivation has an impact on the heritage of the craftsmanship spirit, although the impact is small; it requires concerted actions together with high institutionalization, non-high intuition, and non-high interpretation to achieve its due effect. Additionally, this configuration indicates that the integrative and interactive learning system with craftsmen as the core has no effect on the heritage of the craftsmanship spirit. This configuration covers two cases, and the cases show that craftsmen generally like the skills and knowledge of the master, often think about the gap between themselves and the master, abide by the organization’s laws and regulations, and are relatively familiar with the organizational system but also lack verbal communication, with almost no communication between craftsmen and other colleagues; this mainly manifests as the absence of verbal expression, dialog and cognitive creation with other colleagues, indicating the absence of non-high interpretation in these cases. Moreover, aside from sophisticated institutional rules, craftsmen at work also consider family factors while attaching importance to achieving their own work goals, indicating that in the absence of dialog with other colleagues, craftsmen compensate through the joint action of the high institutional construction of the organization, family motivational incentives and target incentives.

The presence of high institutionalization in configuration H1b also plays a core role, while the presence of non-high family motivation and non-high integration as well as the absence of non-high interpretation play a supporting role. Obviously, the integrative learning in this configuration is alternative to the intuitive learning in the H1a configuration and, together with the three remaining conditions, promote the efficient heritage of the craftsmanship spirit. Further comparison reveals that both integrative learning and intuitive learning are the core learning processes of craftsmen, of which integrative learning emphasizes team collective interactions with craftsmen as the core, while intuitive learning emphasizes the goal orientation, skills and experience dissemination of individual craftsmen. This configuration covers two cases, and one case is shared with H1a. The cases show that first, craftsmen can be independent, start to train their own apprentice craftsmen, and have a certain degree of confidence in their work, with the master allowing the craftsman to handle important tasks; second, craftsmen enjoy work and are good at integrating learning, i.e., they have friendly exchanges with masters and other personnel, work together to complete tasks, are willing to share experiences with other members of the team, and put themselves within a system of mutual help; last, they shoulder the dual responsibilities of family and work, and the affirmation of their work by their family members and the satisfaction of their achievements at work are the driving forces for them to continue to work hard and perfect their already superb skills. In short, a craftsman being entrusted with tasks early in employment and the craftsman’s work and family being valued by the organization (there is a unique incentivization measure in the exampled companies; when an employee shows outstanding performance and becomes a team leader, i.e., assume the master role, the employee’s family was awarded 50,000 yuan) demonstrate the importance of institutional construction while highlighting the necessity of incentivizing craftsman’s family members.

In the H1c configuration, which covers six cases, the presence of high institutionalization is the core condition, while the presence of non-high intuition, non-high interpretation and non-high integration are peripheral conditions. The analysis of this configuration indicates that first, the craftsmen in the cases have high learning and comprehension abilities and fully devote themselves to work, with some craftsmen immersing themselves in work, i.e., “workaholics” (Clark et al., 2014). Second, they do not pay too much attention to things outside of work, including promotion, personal intercourse, etc., and some craftsmen disregard family members’ feelings and exhibit non-prosocial behaviors, leading to frequent work-family conflicts. It is also found that when craftsmen begin their apprenticeship, there is often discord in the mentor-apprentice relationship. However, with time, these craftsmen gradually blend into the team. In short, these craftsmen fully devote themselves to work and do not care about others’ opinions, with strong self-awareness, and they sometimes neglect family and interpersonal relations and thus are typical “work-centered” employees.

(2) Motivation-driven interpretation-led configuration–In configuration H2, the presence of high family motivation and high interpretation plays a central role while that of non-high intuition and non-high integration plays a peripheral role. This configuration covers six cases, and the average work experience of the involved craftsmen is 12.5 years, which is significantly shorter than that of craftsmen in other cases, indicating that craftsmen work mainly for the purpose of “raising a family” and are still in the growth stage in terms of understanding their work, skills, and professionalism. The results of the analysis indicate that the configuration of high family motivation and high interpretation as the core conditions is conductive to the family. Furthermore, for these craftsmen, their immediate family members, relatives and friends are proud of their reputation, job title and pay. Therefore, they can receive support from their family. Meanwhile, apprentices who have gained family support and approval are further motivated, with increased feeling of happiness; they use family members’ opinions, comments, and attitudes as an important criteria for career choices. However, exceptional professional skills often make these craftsmen single-skilled, which not only makes them very difficult to engage in other occupations, but also becomes one of the reasons that the craftsmen can do the same job diligently.

The craftsmen in these cases are good at communicating and building their own social network while humbly asking their masters for advice. They are also willing to establish a self-centered work network, but due to a lack of prestige and experience, their learning behavior remains at the personal level. Overall, craftsmen with motivation-driven interpretation-led configurations attach importance to both family factors and dialog with the outside world, but an excessively flexible working style often makes them not attentive to institutional constructs by not being bound by organizational framework and inertia.

In addition, the configuration of non-efficient heritage of the craftsmanship spirit, i.e., the NH1 configuration, is also analyzed, and the results show that the absence of high family motivation and high institutionalization leads to the emergence of non-efficient heritage of the craftsmanship spirit, which, from the perspective of reverse inhibitory factors, proves the importance of the combined effect of family motivation and institutionalization on the heritage of the craftsmanship spirit.

## Discussion

### Conclusion

The promotion of efficient heritage of the craftsmanship spirit requires a series of combined conditions. Based on social cognitive theory, the inheritance process of craftsmanship spirit is limited to the perspective of the interaction between individual cognition and behavior. This study investigates craftsmen in apprenticeships, validates the fitting relations in the 4I framework of family motivation and organizational learning, and obtains two paths for promoting the efficient heritage of the craftsmanship spirit (containing four configurations). This study comprehensively analyses the condition combinations of the two paths and the cases covered by each path to reveal the black box of the relationship between family motivation and heritage of the craftsmanship spirit.

The use of configurations and fsQCA reveals in the apprenticeship workplace. There are four configurations that can promote the inheritance of apprentice craftsman spirit and can form two fitting paths: institutionalization-led path and motivation-driven interpretation-led path. In the institutionalization-led path, the institutionalized learning all appear in three configurations. When both high family motivation and low explanation appear (H1a, H1b), intuition and integration will have the same effect on efficient inheritance. This shows that an organization’s own management framework, inertia and strategy are not only institutional guarantees in apprenticeship systems but also environmental guarantees for craftsmen to work attentively, without disruption. To cultivate and pass on the craftsmanship spirit, enterprises should strengthen institutional constructs at the organization level. The results of the H1c configuration show that when organizational learning behavior plays a significant role, family motivation is irrelevant. Although this study mainly focuses on fitting family motivation to organizational learning, the irrelevance of family motivation revealed in this configuration can explain the heritage path of the craftsmanship spirit “workaholic” craftsmen from another perspective and reflect the important role of organizational learning behavior in enhancing craftsman’s work attitude and enthusiasm, while verifying the presence of the phenomenon that “reinforcing learning behavior may lead to a lack of family motivation.”

In the motivation-driven interpretation-led path, due to the lack of institutionalized learning, high family motivation and high explanation will become more obvious. They form a new conditional configuration together with intuition and integration as peripheral conditions. In the configuration of non-efficient inheritance, with the absence of interpretation and integration, low family motivation and low institutionalized learning are the core conditions. Together with low intuition, they lead to the result of non-efficient inheritance.

### Theoretical Contribution

In previous studies, family motivation is used as a single influencing factor for active work (Shao et al., 2017; Umrani et al., 2019; Erum et al., 2021), rather than considering family motivation as a configuration. Relative to existing studies, this study examines the fitting relationship between family motivation and organizational learning behavior and has made the following contributions.

First, regarding heritage of the craftsmanship spirit, the most important theoretical contribution of this study proposes a cognitive learning system to explain the inheritance of craftsmanship spirit and to verify a fitting relationship between family motivation and organizational learning. It is well established that family motivation makes work more valuable (Rosso et al., 2010). Based on the above conclusion, this study further verifies a fitting relationship between family motivation and organizational learning, and an important role that plays in promoting the efficient inheritance of craftsmanship spirit. There is a configuration effect in family motivation and 4I (intuition, interpretation, integration, and institutionalization) learning behavior (Grant, 2007). These factors work together to promote the efficient heritage of the craftsmanship spirit in the apprenticeship system.

Second, there are multiple fitting relationships between family motivation and organizational learning behavior, and the theoretical value contained in each of the relationships has opened up a new perspective for the study of the heritage of the craftsmanship spirit. By using the configuration-based fsQCA, this study assesses various combination conditions that promote craftsman’s heritage of the craftsmanship spirit and reveals the effective paths for heritage by craftsmen with different personality characters. The findings of this study help answer the question of how craftsmen with different family motivation levels can efficiently inherit the craftsmanship spirit. Specifically, this study explains not only how craftsmen with high family motivation effectively inherit the craftsmanship spirit but also why craftsmen with low willingness regarding “working for the family” can also inherit the craftsmanship spirit efficiently.

### Management Implications

In the Chinese context of advocating the craftsmanship spirit, the work mode of the apprenticeship system needs to be promoted vigorously. Increasingly more scholars have begun to analyze the heritage of the craftsmanship spirit from the perspective of organizational behavior. This study investigates how family motivation stimulates craftsman’s work enthusiasm, which also has management implications. First, family motivation helps promote attentive and dedicated work by craftsmen. Certainly, traditional incentives for craftsmen, e.g., promotions, rewards, pay raises, etc., can help craftsmen develop better work styles, and other initiatives that focus on rewarding craftsman’s family members, such as “dedicated to work and family,” “work/family balance,” and “company is a family,” should be valued by the company and implemented. Second, humanistic care centered on family members should be strengthened. A company’s assistance in medical care, schooling, and employment of craftsman’s family members could enable craftsmen to be more dedicated to work and help cultivate loyalty to the company. Third, enterprises should refine policies based on the personalities of different craftsmen and accordingly implement categorized management. The learning trajectory and attitude toward work of craftsmen with different personalities or in different work environments differ. For example, for “workaholic” craftsmen, the motivation of work itself should be strengthened, and for craftsmen with strong family motivation, incentives of recognition, material rewards or other necessary help for family members should be strengthened.

### Limitations

This study also has some limitations, and some conclusion need further verification. For example, when analyzing craftsman’s family motivation and organizational learning, this study did not investigate mentoring relationships and affection between the master and apprentice, but in the apprenticeship system, the role of the master may be crucial in determining a craftsman’s family motivation and increase his or her organizational learning ability. Therefore, in the study of craftsman’s family motivation and learning abilities, the inclusion of the master-apprentice relationship as a moderating variable is an issue worthy of further investigation. In addition, when examining the role of family motivation and organizational learning ability in the heritage of the craftsmanship spirit, personality should not be ignored. The configuration analysis results of this study have revealed the influencing pattern of the individual personality of each craftsman on family motivation, and with a weakened family motivation triggered by each individual’s personality, individual personality can promote the efficient heritage of the craftsmanship spirit. Therefore, the space for the study of the relationship between family motivation and the craftsmanship spirit can be further expanded by studying the underlying micromechanisms.

## Data Availability Statement

The original contributions presented in the study are included in the article/supplementary material, further inquiries can be directed to the corresponding author.

## Ethics Statement

Ethical review and approval was not required for the study on human participants in accordance with the local legislation and institutional requirements. Written informed consent for participation was not required for this study in accordance with the national legislation and the institutional requirements.

## Author Contributions

GC performed conceptualization and drafted manuscript. GC and JD performed methodology and formal analysis. RS performed investigation. LL proposed amendments and references. XM participated in its design and coordination. All authors read and approved the final manuscript.

## Conflict of Interest

The authors declare that the research was conducted in the absence of any commercial or financial relationships that could be construed as a potential conflict of interest.

## Publisher’s Note

All claims expressed in this article are solely those of the authors and do not necessarily represent those of their affiliated organizations, or those of the publisher, the editors and the reviewers. Any product that may be evaluated in this article, or claim that may be made by its manufacturer, is not guaranteed or endorsed by the publisher.
